# Activation of the *CDK7* Gene, Coding for the Catalytic Subunit of the Cyclin-Dependent Kinase (CDK)-Activating Kinase (CAK) and General Transcription Factor II H, by the Trans-Activator Protein Tax of Human T-Cell Leukemia Virus Type-1

**DOI:** 10.3390/genes15081080

**Published:** 2024-08-15

**Authors:** Mashiro Shirasawa, Rinka Nakajima, Yaxuan Zhou, Lin Zhao, Mariana Fikriyanti, Ritsuko Iwanaga, Andrew P. Bradford, Kenta Kurayoshi, Keigo Araki, Kiyoshi Ohtani

**Affiliations:** 1 Department of Biomedical Sciences, School of Biological and Environmental Sciences, Kwansei Gakuin University, 1 Gakuen Uegahara, Sanda 669-1330, Hyogo, Japan; idl05439@kwansei.ac.jp (M.S.); hnj51097@kwansei.ac.jp (R.N.); gtk53096@kwansei.ac.jp (Y.Z.); hsj19688@kwansei.ac.jp (M.F.); 2 Department of Obstetrics and Gynecology, University of Colorado School of Medicine, Anschutz Medical Campus, 12700 East 19th Avenue, Aurora, CO 80045, USA; ritsuko.iwanaga@cuanschutz.edu (R.I.); andy.bradford@cuanschutz.edu (A.P.B.); 3 Division of Molecular Genetics, Cancer Research Institute, Kanazawa University, Kakuma-machi, Kanazawa 920-1192, Ishikawa, Japan; kentakurayoshi@staff.kanazawa-u.ac.jp; 4 Department of Morphological Biology, Ohu University School of Dentistry, 31-1 Misumido Tomitamachi, Koriyama 963-8611, Fukushima, Japan; keigoaraki.res@gmail.com

**Keywords:** HTLV-1, Tax, ATL, trans-activation, CDK7

## Abstract

Human T-cell leukemia virus type-1 (HTLV-1) is the etiological agent of adult T-cell leukemia (ATL). The trans-activator protein Tax of HTLV-1 plays crucial roles in leukemogenesis by promoting proliferation of virus-infected cells through activation of growth-promoting genes. However, critical target genes are yet to be elucidated. We show here that Tax activates the gene coding for cyclin-dependent kinase 7 (CDK7), the essential component of both CDK-activating kinase (CAK) and general transcription factor TFIIH. CAK and TFIIH play essential roles in cell cycle progression and transcription by activating CDKs and facilitating transcriptional initiation, respectively. Tax induced *CDK7* gene expression not only in human T-cell lines but also in normal peripheral blood lymphocytes (PHA-PBLs) along with increased protein expression. Tax stimulated phosphorylation of CDK2 and RNA polymerase II at sites reported to be mediated by CDK7. Tax activated the CDK7 promoter through the NF-κB pathway, which mainly mediates cell growth promotion by Tax. Knockdown of CDK7 expression reduced Tax-mediated induction of target gene expression and cell cycle progression. These results suggest that the *CDK7* gene is a crucial target of Tax-mediated trans-activation to promote cell proliferation by activating CDKs and transcription.

## 1. Introduction

Human T-cell leukemia virus type 1 (HTLV-1) is the first retrovirus identified in humans that is an etiological agent of adult T-cell leukemia (ATL) [1,2,3,4]. HTLV-1 infection also causes HTLV-1-associated myelopathy (HAM)/tropical spastic paraparesis (TSP) and other inflammatory diseases [4,5,6,7,8]. The HTLV-1 genome has a unique *pX* region, which codes for several regulatory proteins such as trans-activator of the *pX* region (Tax) and Rex [3]. Among these regulatory proteins, Tax plays essential roles in transformation of HTLV-1 infected cells. Tax is a multifunctional protein, which modulates transcription to activate viral and cellular gene expression, promotes cell cycle progression, suppresses apoptosis and induces genomic instability [3,4]. Tax facilitates viral replication by functioning as a transcriptional activator of its own promoter via the 5′ LTR [9,10]. Tax also trans-activates many cellular genes involved in cell proliferation and survival, and it is thought to play crucial roles in transformation of the virus-infected cells [3,4,11]. Viral vector-mediated introduction of Tax in normal peripheral blood T-cells immortalized the cells, depending on the T-cell growth factor IL-2 [12,13], and Tax expression phenotypically transformed immortalized rat fibroblasts [14,15,16]. Tax-transgenic mice developed mesenchymal tumors, adenoma and lymphoma/leukemia depending on the promoter used [17,18,19,20]. These observations support the notion that Tax plays central roles in leukemogenesis induced by HTLV-1 infection. Indeed, Tax has the ability to promote cell cycle progression [21,22,23], which may contribute to aberrant proliferation of virus-infected cells. However, too much Tax activity induces cellular senescence, and HTLV-1 basic zipper protein (HBZ), coded in the anti-sense strand, counteracts many functions of Tax to allow persistent viral infection [24,25,26,27,28,29]. Target genes of Tax identified to date are those coding for growth factors, cytokines and their receptors, growth signal transducers, transcription factors, cell cycle regulators, cell attachment molecules and others. Among these, Tax-induced cell cycle progression is paralleled by induction of cell cycle regulatory genes, such as *CCND2* and *cyclin-dependent kinase* (*CDK*) *6*, whose knockdown suppressed Tax-mediated cell cycle progression [30]. Induction of CCND2 and CDK6 inactivates the tumor suppressor pRB and leads to activation of the transcription factor E2F, especially E2F1, which is essential for cell cycle progression [23,31]. These observations suggest that induction of cell cycle regulatory genes by Tax is crucial for Tax-induced cell cycle progression. Tax interacts with variety of cellular factors to activate at least three transcription factors: cAMP responsive element-binding factor (CREB), nuclear factor (NF)-κB and serum-responsive factor (SRF), to activate target genes [3]. Accumulating evidence indicates that Tax-induced activation of NF-κB is critical for immortalization of T-cells [11,32,33]. Consistent with this, the ability of Tax to promote cell cycle progression correlates with that to activate NF-κB [23,30,31], and Tax activates the CCND2 promoter primarily through the NF-κB pathway [34]. These observations underscore the importance of Tax-mediated activation of target genes and CDKs for consequent activation of E2F and cell cycle progression. However, the repertoire of Tax target genes, especially those necessary for leukemogenesis, is not known in detail [11].

To better understand the spectrum of Tax targets and explore those critical for promotion of cell proliferation, we identified new targets of Tax-mediated trans-activation in our recent report [35], using Kit 225 cells [36]. Kit 225 is an IL-2-dependent human T-cell line, which can be synchronized in the resting state by culturing in the absence of IL-2, without significant cell death, and will re-enter the cell cycle upon addition of IL-2 [23]. Moreover, expression of Tax can promote cell cycle progression in IL-2-starved Kit 225 cells [23]. Asynchronously growing Kit 225 cells were infected with Tax-expressing recombinant adenovirus or control virus, cultured for two days in the absence of IL-2 and harvested. Gene expression profiles were examined by DNA microarray, and genes that showed higher expression in Tax-expressing cells than control cells were determined. The top 300 genes induced by Tax were reported, in order of magnitude of induction [35]. These included 20 genes that are known target genes of Tax, indicating the validity of the screening. In this study, among the newly identified genes, we focused on the *CDK7* gene (Rank 83 in Supplementary Table S1, 2.1-fold induction by Tax) [35], which is not a previously identified Tax target, since CDK7 plays essential roles in cell cycle progression and the initiation of transcription.

CDK7 is the catalytic component of CDK-activating kinase (CAK). CDKs involved in cell cycle progression are mainly CDK4/6, which functions in the G1 phase together with CCND, CDK2 acting in the late G1 to S phases together with CCNE and CCNA, and CDK1 functioning in the G2 to M phases together with CCNA and CCNB, as partners. These CDKs require not only the cyclin partners but also phosphorylation at the activation segment T-loop for activation, which is mediated by CDK7 [37]. CAK is composed of CDK7, CCNH and Menage a trois homolog 1 (MAT1), as essential components [38,39]. Thus, CDK7 plays essential roles in progression of the cell cycle by activating the CDKs involved in cell cycle progression.

CAK is also an essential component of transcription factor II H (TFIIH), which is required for initiation of transcription in the preinitiation complex, at promoters of genes transcribed by RNA polymerase II. RNA polymerase II recruited to the transcriptional start site is paused by anchoring of the C-terminal domain of RBP1, the largest subunit of RNA polymerase II. The human C-terminal domain of RBP1 contains 52 repeats of a heptad amino acid sequence (Tyr-Ser-Pro-Thr-Ser-Pro-Ser). To start transcription, phosphorylation of the C-terminal domain of RNA polymerase II (CTD) is required to release from anchoring. CDK7 mediates this phosphorylation at the Ser5 and Ser7 positions of the heptad sequence [40,41], thereby playing essential roles in transcriptional initiation. Since the basic mechanisms of Tax-mediated promotion of cell cycle progression are activation of target genes and CDKs, the *CDK7* gene could be a crucial target of Tax.

It is generally accepted that activity of CDKs is regulated by expression of their partner cyclins. Since Tax induced expression of the *CDK7* gene, we examined the roles of CDK7 in Tax-mediated activation of target genes and cell cycle progression in relation to its partner CCNH. Our results show that Tax induced expression of CDK7 but not its partner CCNH. In spite of this, Tax enhanced phosphorylation of CDK2 and the C-terminal domain of RNA polymerase II, which are reported to be mediated by CDK7. Moreover, downregulation of Tax-mediated CDK7 induction by shRNA suppressed induction of target gene expression and cell cycle progression induced by Tax. These observations suggest a unique functional interaction of CDK7 and CCNH, compared to that of other canonical CDKs and cyclins, in the control of its activity, and underscore the *CDK7* gene as a critical target of Tax in trans-activation of target genes and promotion of cell cycle progression.

## 2. Materials and Methods

### 2.1. Cell Culture

HTLV-1-unrelated IL-2-dependent human T-cell line Kit 225 [36] was maintained in RPMI 1640 medium containing 10% fetal calf serum (FCS) and 1 nM IL-2 (Ajinomoto, Yokohama, Japan). HTLV-1-unrelated human T-cell lines Jurkat [42], CCRF-CEM [43] and MOLT-4 [44] and HTLV-1-infected human T-cell lines MT-1 [1], TL-Om1 [45], HuT 102 [46], MT-2 [47], MT-4 [48] and TL-Su [49] were maintained in RPMI 1640 medium containing 10% FCS. Human kidney epithelial cell line HEK-293 [50] was maintained in Dulbecco’s modified Eagle’s medium containing 10% FCS.

### 2.2. Plasmids

pCDK7(-1726)-Luc was generated by cloning the –1726 to +47 region of CDK7 promoter into pGL3-Basic (Promega) using the *Mlu*I and *Bgl*II sites. pCDK7US-Luc and pCDK7DS-Luc were generated by cloning the −2546 to −1732 region and +51 to +815 region containing the 1st exon to 1st intron of the *CDK7* gene into pGL3-Promoter (Promega, Madison, WI, USA) using the *Bgl*II and *Mlu*I sites, respectively. pENTR-shCDK7-1, pENTR-shCDK7-2, pENTR-shp65-1, pENTR-shp65-2 and pENTR-shp65-3 were generated by replacing the control sequence of pENTR-shCon with shRNA sequences against CDK7 and p65 (target sequences are listed in RNA interference). pENTR-shCon has an expression cassette subcloned from pSilencer 2.0-U6 (Ambion, Austin, TX, USA) in pENTR/D-TOPO (Invitrogen, Carlsbad, CA, USA). pE2WTx4-Luc, pκB-Luc, pWT-Luc, pCArG-Luc, pCycD2-Luc(-1624), pMT2-Tax, pHβAPr-1-neo, pCMV-β-gal, pENTR-shCon, pENTR-shp100-1 and pENTR-shp100-2 were described previously [23,30].

### 2.3. Transfection and Reporter Assay

Expression plasmids and reporter plasmids were introduced into asynchronously growing Kit 225 or Jurkat cells using the DEAE-dextran method as described previously [51]. pCMV-β-gal was included as an internal control. Plasmids were suspended in 1 mL of RPMI 1640 medium, and 150 μL of DEAE-dextran solution (1M Tris-HCl (pH 7.4), 5 mg/mL DEAE-dextran) was added. Cells (5.0 × 10^6^) were suspended in 1 mL of RPMI 1640 medium, added into the cocktail and incubated for 30 min at 37 °C. The cells were mixed with 3 mL of RPMI 1640 medium containing 10 U/mL Na heparin to neutralize DEAE-dextran. After washing with RPMI 1640 medium, the cells were cultured in 20 mL of RPMI 1640 medium containing 10% FCS in the absence of IL-2 for 2 or 3 days, and luciferase activities were measured as described previously [52]. The cells were harvested and lysed in 100 μL of passive lysis buffer (Promega, Madison, WI, USA). Supernatants were collected after centrifugation, and 10 μL was used for the luciferase assay and 50 μL for the β-galactosidase assay. Luciferase activity was measured using a luciferase assay system (Promega, Madison, WI, USA) and a luminometer Lumat LB 9507 (Berthold Technologies, Bad Wildbad, Germany). β-galactosidase activity was measured in 500 μL of Z-buffer (60 mM Na_2_HPO_4_, 40 mM NaH_2_PO_4_, 10 mM KCl, 1 mM MgSO_4_, 40 mM 2-mercaptoethanol) containing 4 mg/mL 2-nitrophenyl-β-D-galactopyranoside (ONPG) at 37 °C. The reaction was stopped by the addition of 250 μL of 1 M Na_2_CO_3_ so that the absorbance at 410 nm fell within 0.1 to 1.0. Luciferase activity was adjusted by the β-galactosidase activity as an internal control. All assays were performed in triplicate and the results are presented as means ± SD.

### 2.4. Recombinant Adenovirus

Recombinant adenovirus expressing Tax (AxCAIY-Tax) and control virus (Ad-IWI), and Ad-shCon were described previously [23,30]. Ad-shCDK7-1, Ad-shCDK7-2, Ad-shp65-1, Ad-shp65-2 and Ad-shp65-3 were generated from pENTR-shCDK7-1, pENTR-shCDK7-2, pENTR-shp65-1, pENTR-shp65-2 and pENTR-shp65-3, respectively, using a ViraPower Adenoviral Expression System (Invitrogen, Carlsbad, USA) according to the supplier’s protocol. The viruses were expanded in HEK-293 cells and purified using a CsCl density gradient. The titer of the purified viruses was determined by serial dilution and infection of HEK-293 cells, followed by staining the *E2* gene product with a specific antibody raised against synthetic peptides in rabbits (a kind gift from Dr. M. Ikeda, Tokyo Medical and Dental University). Infection of cells with recombinant adenovirus was achieved in 1 mL of RPMI 1640 medium at the indicated multiplicity of infection (MOI) for 1 h at 37 °C.

### 2.5. RNA Interference

Target sequences of shRNA against CDK7 (shCDK7), p65 (shp65) and p100 (shp100) were as follows:

shCDK7-1: 5′-AGCCTACATGTTGATGACTT-3′;
shCDK7-2: 5′-AGGAGCAATCAAATCCAGC-3′;
Nucleotides 435–453 and 1043–1061 of human Cdk7 cDNA (GenBank Accession No. NM_001799), respectively.
shp65-1: 5′-TGAGGCTATAACTCGCCTA-3′;
shp65-2: 5′-GGATTGAGGAGAAACGTAA-3′;
shp65-3: 5′-CTGTTCCCCCTCATCTTCC-3′;
Nucleotides 1575–1593, 974–992 and 94–112 of human RelA cDNA (GenBank Accession No. 021975), respectively.
shp100-1: 5′-AAGATGACATTGAGGTTCGGT-3′;
shp100-2: 5′-CAACTGAAACGCAAGCGAGGA-3′;
Nucleotides 928–948 and 1086–1106 of human p100 cDNA (GenBank Accession No. 002502), respectively.

### 2.6. Quantitative Reverse Transcription (qRT)-PCR

qRT-PCR was conducted as described previously [53]. Total RNA was isolated using Isogen II (Nippon Gene). First-strand cDNA was synthesized using a 1st Strand cDNA Synthesis Kit for RT-PCR [AMV] (Roche) using an oligo(dT) primer. Quantitative PCR was carried out using KAPA SYBR qPCR Mix (KAPA Biosystems) and a Thermal Cycler Dice Real Time System Single (TaKaRa). Specific primer sets used were as follows:

*CDK7*
Fw: 5′-GTGCAGCAGGAGACGACTTACTA-3′;
Rv: 5′-GGCCTCTGTTCTTTTCCTTTTTAT-3′.
*CCNH*
Fw: 5′-TTGCCCTGACTGCCATTT-3′;
Rv: 5′-GACAGGCAAGTTCTGTTCTCT-3′.
*CDK9*
Fw: 5′-TGGGCTGTTGAGCAATGT-3′;
Rv: 5′-CCCTATGCAGGATCTTGTTTCT-3′.
*CCNT*
Fw: 5′-CGAGGCTGCCAAGAAAACAAAAG-3′;
Rv: 5′-CCCGGCAACATCTCCACACTG-3′.
*CDK6*
Fw: 5′-GTAATCGTGTCTGTGTTGAG-3′;
Rv: 5′-TCTGCACCCGCACGCGTTC-3′.
*CCND2*
Fw: 5′-CTGTGTGCCACCGACTTTAGTT-3′;
Rv: 5′-GATGGCTGGCCCACACTTC-3′.
*GAPDH*
Fw: 5′-GGAGTCCACTGGCGTCTTCA-3′;
Rv: 5′-CAGGGGCCATCCACAGTCTT-3′.

### 2.7. Fluorescence-Activated Cell Sorting (FACS) Analysis

FACS analysis of the cell cycle distribution was performed as described previously [53]. Cells were fixed with 70% ethanol at −20 °C overnight, and DNA was stained with propidium iodide (PI) solution (50 μg/mL PI, 50 μg/mL RNase A, 0.5% bovine serum albumin (BSA) and 15 mM NaN_3_ in PBS) for more than 30 min at room temperature. DNA contents of the cells were analyzed with a FACSCalibur (Becton Dickinson, Franklin Lakes, NJ, USA).

### 2.8. Immunoblot Analysis

Immunoblot analysis was performed as described previously [31]. For protein extraction, the cells were suspended in 5 times the cell pellet volume of RIPA buffer (150 mM NaCl, 1% NP-40, 0.5% deoxycholic acid (DOC), 0.1% sodium dodecyl sulfate (SDS), 50 mM Tris-HCl (pH 8.0)) containing complete protease inhibitor cocktail (Roche, Basel, Switzerland). For immunoblotting with a phosphorylation site-specific antibody, PhosSTOP (Roche, Basel, Switzerland) was also added. The mixture was left on ice for 30 min, and supernatant was collected after centrifugation. The protein concentration was measured using a Protein Assay Kit (BIO-RAD, Hercules, CA, USA). The absorbance was measured at 595 nm within the range of 0.1 to 1.0. The protein concentration was deduced from a standard curve made by using BSA. The same amounts of protein samples (30~50 μg) were electrophoresed on an SDS polyacrylamide gel at 200 V for 2 h. Separated proteins were transferred to a PVDF membrane (Paul) using a TRANS-BLOT SD SEMI-DRY TRANSFER CELL (BIO-RAD, Hercules, CA, USA) at 25 V for 30 min. The membrane was blocked using 5% skim milk (BD Biosciences, Sparks, NV, USA) in TTBS (0.1% Tween in TBS) for 1 h, and reacted with a first antibody in 0.5% skim milk in TTBS overnight at 4 °C. The membrane was washed 3 times for 20 min with TTBS, and reacted with a secondary antibody for 1 h at room temperature. The antibodies used were anti-Tax antibody Lt-4 (ascites form, 1:1000) [54], anti-CDK7 (556345, BD Biosciences, Sparks, NV, USA, 1:500), anti-phosphorylated CDK2 (Thr160) (P-CDK2, T160, 2561S, Cell Signaling Technology, Danvers, MA, USA, 1:500), anti-CDK2 (sc-163-G, Santa Cruz Biotechnology, Dallas, TX, USA, 1:500), anti-phosphorylated C-terminal domain of RNA polymerase II (Phospho-Rpb1 CTD (Ser5) (D9N5I) Rabbit mAb, #13523, Cell Signaling Technology, 1:500), anti-C-terminal domain of RNA polymerase II (Rpb1 CTD (4H8) Mouse mAb, #2629, Cell Signaling Technology, 1:500), anti-p65 (sc-372, Santa Cruz Biotechnology, Danvers, USA, 1:500), anti-α-tubulin (DM1A, Calbiochem, 1:1000), anti-β-actin (A1978, SIGMA, St. Louis, MO, USA, 1:2000), peroxidase-conjugated anti-mouse IgG (NA9310, Amersham, UK, 1:5000), peroxidase-conjugated anti-rabbit IgG (NA934, Amersham, UK, 1:5000) and peroxidase-conjugated anti-goat IgG (sc-2020, Santa Cruz Biotechnology, Dallas, USA, 1:5000). After similarly washing 3 times with TTBS, the chemiluminescent signals were detected using LAS4000 (GE Healthcare, Chicago, IL, USA) after treating the membrane with ImmunoStar LD (Fujifilm, Tokyo, Japan), and quantified by ImageJ (version 1.51s) (NIH).

### 2.9. Statistical Analysis

Reporter assays and qRT-PCR experiments were conducted in triplicate. Data were presented as means ± SD. Statistical comparisons were made using Student’s *t*-test and Bonferroni correction. A *p* value < 0.05 was considered significant.

## 3. Results

### 3.1. Tax Induces CDK7 Gene Expression in Human T-Cells

**Figure 1:**
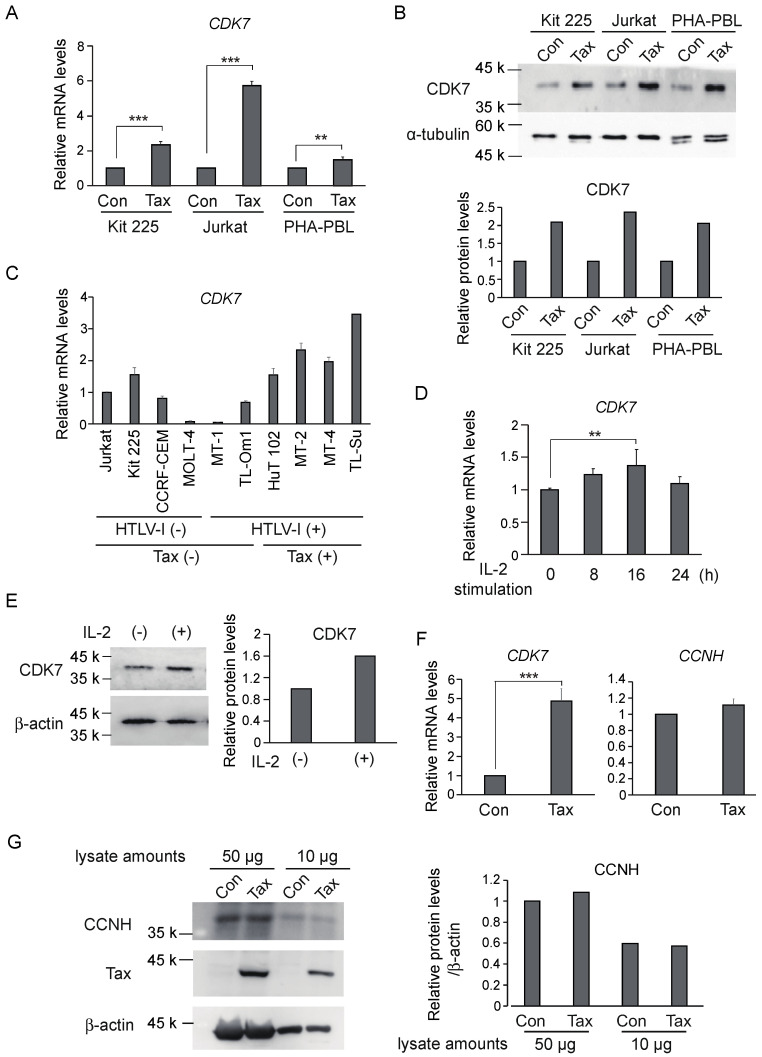
(**A**) Asynchronously growing Kit 225, Jurkat and PHA-PBLs were infected with Tax-expressing adenovirus or control virus at an MOI of 100, further cultured for 48 h in the absence of IL-2 and harvested. Expression levels of *CDK7* mRNA were examined by qRT-PCR, adjusted by those of *GAPDH* as an internal control. Relative expression levels are presented as that of the control virus as 1. **: 0.01 ≤ *p* < 0.05, ***: *p* < 0.01. (**B**) Under the same condition, expression levels of CDK7 protein were examined by Western blot analysis (upper panel). α-tubulin was used as an internal control. Intensities of the bands were measured by ImageJ, and the levels of CDK7 expression were adjusted by those of α-tubulin (lower panel). (**C**) The expression levels of *CDK7* mRNA were examined by qRT-PCR in the indicated cell lines, adjusted by those of *GAPDH* as an internal control. Relative expression levels are presented. (**D**) Kit 225 cells were starved of IL-2 for 2 days, re-stimulated by IL-2 for the indicated times and harvested. The levels of *CDK7* mRNA were examined by qRT-PCR, adjusted by those of *GAPDH* as an internal control. Fold inductions by IL-2 are presented. **: 0.01 ≤ *p* < 0.05. (**E**) Under the same conditions, CDK7 protein expression was examined at 16 h after IL-2 stimulation by Western blot analysis (upper panel). β-actin was used as an internal control. Intensities of the bands were measured by ImageJ, and the levels of CDK7 expression were adjusted by those of β-actin (lower panel). (**F**) Asynchronously growing Kit 225 cells were infected with Tax-expressing adenovirus or control virus at an MOI of 100, further cultured for 48 h in the absence of IL-2 and harvested. Expression levels of *CDK7* and *CCNH* mRNAs were examined by qRT-PCR, adjusted by those of *GAPDH* as an internal control. Relative expression levels are presented as that of the control virus as 1. ***: *p* < 0.01. (**G**) Under the same conditions, the level of CCNH protein was examined by Western blot analysis with two different amounts of sample (left panel). β-actin was used as an internal control. Intensities of the bands were measured by ImageJ, and the levels of CDK7 expression were adjusted by those of β-actin (right panel).

We first examined Tax regulation of *CDK7* gene expression in the human T-cell line Kit 225. We expressed Tax in Kit 225 cells by recombinant adenovirus-mediated gene transfer, and the cells were starved of IL-2 for 2 days prior to harvesting. The gene transduction efficiency of Kit 225 cells by recombinant adenovirus is about 65% [23]. *CDK7* gene expression was examined by qRT-PCR in the entire infected cell population. Expression of *CDK7* was induced by about 2.5-fold, confirming that Tax stimulates expression of the *CDK7* gene in Kit 225 cells (Figure 1A). Tax can promote cell cycle progression in IL-2-starved Kit 225 cells [23]; thus, Tax-mediated induction of *CDK7* gene expression could be either a direct effect of Tax or an indirect consequence of cell cycle progression. To distinguish these possibilities, we examined whether Tax induced *CDK7* gene expression in a human T-cell line, Jurkat, which is not dependent on IL-2 for cell growth and cannot be arrested by deprivation of growth factors. Tax induced expression of the *CDK7* gene by about 5.8-fold (Figure 1A), suggesting that Tax-mediated induction of the *CDK7* gene expression is independent of cell cycle progression and, moreover, that this effect is not cell line specific. Using a similar approach, we also examined whether Tax could induce *CDK7* gene expression in normal human T-cells, since Tax can induce cell cycle progression in phytohemagglutinin-stimulated peripheral blood lymphocytes (PHA-PBLs) [30]. The gene transduction efficiency of PHA-PBLs by recombinant adenovirus is about 45% [30]. Tax enhanced expression of the *CDK7* gene by about 1.5-fold in PHA-PBLs (Figure 1A), indicating that Tax induces *CDK7* gene expression not only in T-cell lines but also in normal T-cells. 

We next examined whether the Tax-induced expression of the *CDK7* gene resulted in a corresponding increase in CDK7 protein expression, by Western blot analysis in the entire cell population. Levels of CDK7 protein were clearly enhanced, not only in Kit 225 and Jurkat cells but also in PHA-PBLs (Figure 1B), indicating that Tax induces CDK7 protein expression in human T-cells.

We also examined the levels of *CDK7* gene expression in HTLV-1-unrelated and HTLV-1-infected human T-cell lines to see whether endogenous expression of Tax correlates with enhanced expression of the *CDK7* gene. Tax-expressing cell lines HuT 102, MT-2, MT-4 and TL-Su showed higher expression of the *CDK7* gene compared to Tax-non-expressing cell lines Jurkat, CCRF-CEM, MOLT-4, MT-1 and TL-Om1 except Kit 225 (Figure 1C), suggesting that Tax expression correlates with higher *CDK7* gene expression. Taken together, these results suggest that Tax induces *CDK7* gene expression human T-cells.

Tax interacts with and modulates growth signal transduction pathways, thereby promoting autonomous cell proliferation in the absence of exogenous growth stimulation. Thus, expression of Tax target genes is usually induced by growth stimulation, suggesting that *CDK7* gene expression may be similarly enhanced. To explore this possibility, we examined expression of the *CDK7* gene in response to IL-2 stimulation of IL-2-starved Kit 225 cells. Although the degree of induction was less than that by Tax, IL-2 treatment significantly induced *CDK7* gene expression, in a time-dependent manner (Figure 1D). IL-2 simulation also increased CDK7 protein expression (Figure 1E). These observations suggest that *CDK7* gene expression is induced in response to growth stimulation in T-cells. 

It is generally accepted that CDKs are expressed relatively constitutively and that their activities are regulated by cyclic induction and destruction of their cyclin partners. Since Tax dramatically increased expression of the *CDK7* gene, we examined whether Tax also induced expression of CCNH, the partner of CDK7. Asynchronously growing Kit 225 cells were infected with Tax-expressing adenovirus or control virus, further cultured in the absence of IL-2 for 2 days and harvested. Expression of the *CCNH* gene was examined by qRT-PCR, along with that of the *CDK7* gene. Unexpectedly, expression of the *CCNH* gene was not significantly impacted by Tax, whereas that of the *CDK7* gene was induced by about 5-fold (Figure 1F). To confirm that expression of *CCNH* was not significantly induced by Tax, we also examined the CCNH protein levels by Western blot analysis. No significant enhancement of CCNH protein level was observed upon Tax expression (Figure 1G). These observations suggest that although Tax significantly induces *CDK7* gene expression, it scarcely induces *CCNH* gene expression in Kit 225 cells.

### 3.2. Tax Induces Phosphorylation of CDK2 and C-Terminal Domain of RNA Polymerase II at the Sites Phosphorylated by CDK7

**Figure 2:**
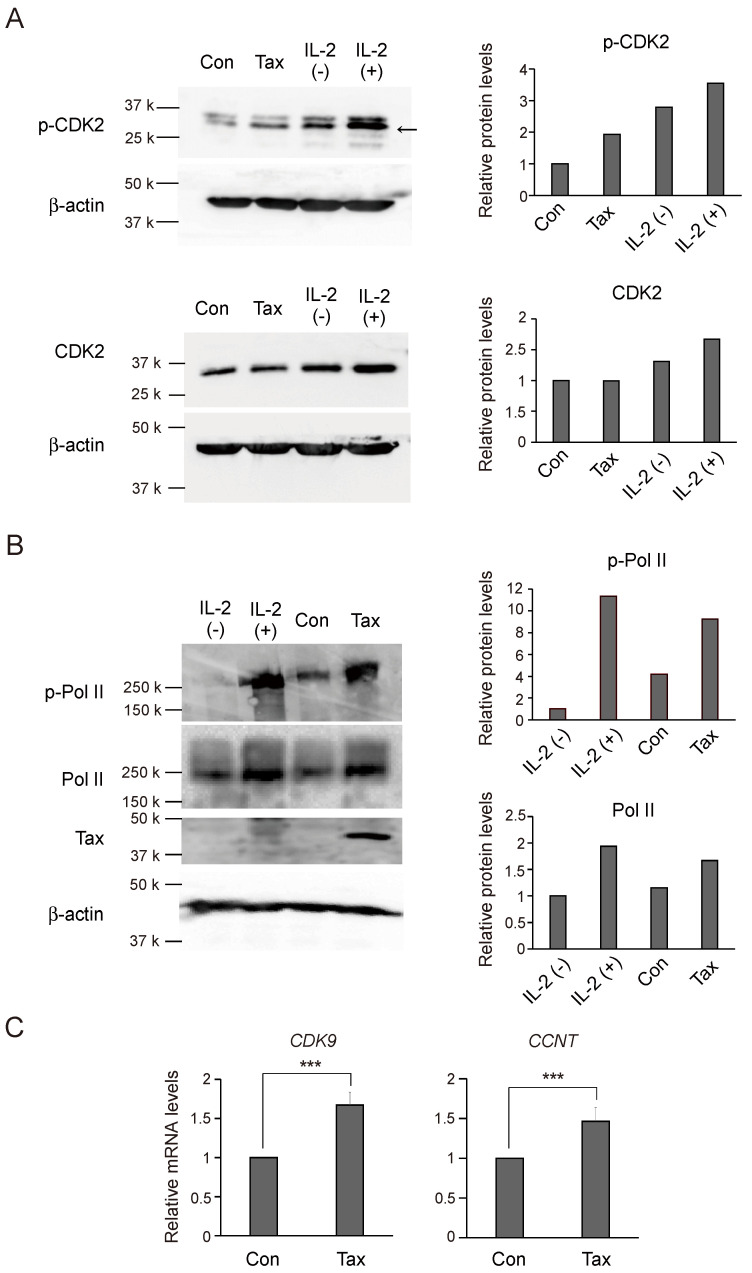
(**A**) Asynchronously growing Kit 225 cells were infected with Tax-expressing adenovirus or control virus at an MOI of 100, further cultured for 48 h in the absence of IL-2 and harvested. IL-2 stimulation was used as a positive control. Kit 225 cells were starved of IL-2 for 2 days, re-stimulated with IL-2 for 16 h and harvested. The levels of phosphorylated CDK2 (p-CDK2) and total CDK2 (CDK2) were examined by Western blot analysis (left panels). β-actin was used as an internal control. The intensities of p-CDK2 and CDK2 were measured by ImageJ and adjusted by those of β-actin (right panels). (**B**) Under the same conditions, the levels of phosphorylated CTD of RNA polymerase II (p-PolII) and total CTD of RNA polymerase II (PolII) were examined by Western blot analysis (left panels). β-actin was used as an internal control and expression of Tax was also confirmed. The intensities of p-PolII and total PolII were measured by ImageJ and adjusted by those of β-actin (right panels). (**C**) Under the same conditions as in Figure 1F, expression levels of *CDK9* and *CCNT* mRNAs were examined by qRT-PCR, adjusted by those of *GAPDH* as an internal control. Relative expression levels are presented as that of the control virus as 1. ***: *p* < 0.01.

To explore whether enhanced expression of CDK7 increases its activity in the absence of alterations in CCNH expression, we explored whether Tax enhances phosphorylation of CDK7 targets. For this purpose, we first examined whether Tax enhances phosphorylation of CDK2, which is involved in the transition from G1 to S phase and passage through S phase, in the cell cycle. Asynchronously growing Kit 225 cells were infected with Tax-expressing virus or control virus, cultured for 2 days in the absence of IL-2 and harvested. Phosphorylation of CDK2 at T160, which is known to be mediated by CDK7, was examined by Western blot analysis using a phosphorylation site-specific antibody. IL-2 stimulation served as a positive control. Tax clearly enhanced phosphorylation of CDK2 (Figure 2A, p-CDK2). 

We used a similar approach to determine whether Tax enhances phosphorylation of the C-terminal domain of RNA polymerase II (CTD), at Ser5 in heptad repeats, which is mediated by CDK7. Tax clearly enhanced phosphorylation of CTD (Figure 2B). These results suggest that expression of Tax enhances phosphorylation of CDK2 and CTD concomitant with enhanced expression of CDK7.

RNA polymerase II released from anchoring by phosphorylation of S5 (and possibly S7) of CTD, catalyzed by CDK7, still pauses at promoter proximal sites, and release requires phosphorylation of S2 of CTD by CDK9/CCNT [55]. We thus examined whether Tax enhanced expression of the *CDK9* and *CCNT* genes to facilitate transcription by releasing polymerase II from pausing. Expression levels of both genes were similarly examined as that of *CDK7* in Kit 225 cells. Tax significantly enhanced expression of both *CDK9* and *CCNT*, although to a lesser extent than *CDK7* (Figure 2C).

### 3.3. Tax Activates CDK7 Promoter in Kit 225 Depending on the NF-κB Pathway but Not in Jurkat

**Figure 3:**
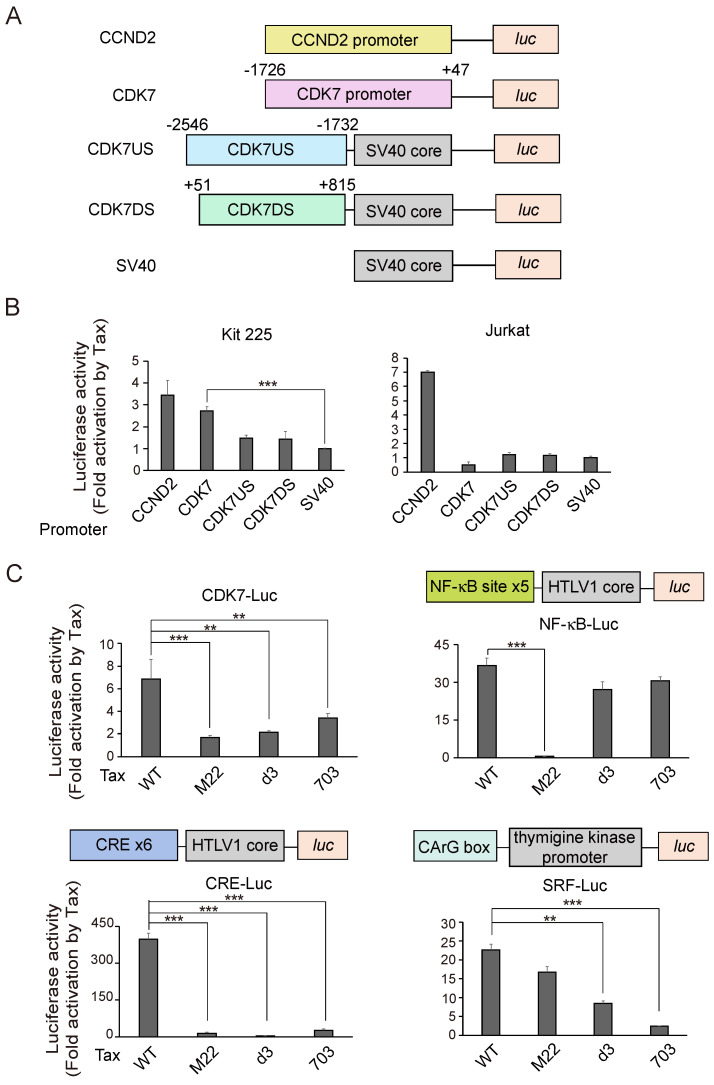
(**A**) Schematic presentation of reporter plasmids. (**B**) Kit 225 or Jurkat cells were transfected with indicated reporter plasmids along with Tax expression vector or control vector, cultured for 2 days in the absence of IL-2 and harvested. Luciferase activities were measured and normalized by those of β-galactosidase as an internal control. Fold activations by Tax are presented. ***: *p* < 0.01. (**C**) Ability of Tax mutants M22, d3 and 703 was similarly examined for activation of CDK7 promoter in Kit 225 cells. Ability of the Tax mutants to activate the NF-κB, CREB and SRF pathways was confirmed using artificial promoters containing tandem repeats of each binding site. **: 0.01 ≤ *p* < 0.05, ***: *p* < 0.01.

Tax induced expression of the *CDK7* gene not only in IL-2-starved Kit 225 cells but also in asynchronously growing Jurkat cells. To analyze the molecular mechanism of Tax-mediated induction of *CDK7* gene expression, we examined whether Tax activated the CDK7 promoter. For this purpose, we cloned the −1726 to +47 region of the CDK7 promoter into a luciferase reporter plasmid and examined whether Tax activated this CDK7 promoter construct in Kit 225 cells. Kit 225 cells were transfected with the CDK7 reporter plasmid, along with Tax-expression vector or control plasmid, and the cells were cultured in the absence of IL-2 for 2 days and harvested. Tax activated the CDK7 promoter by about 2.7-fold (Figure 3B, left panel), suggesting that induction of *CDK7* gene expression by Tax in Kit 225 cells is mediated, at least in part, at the level of transcription. We also examined whether Tax could activate the CDK7 promoter in asynchronously growing Jurkat cells. Unexpectedly, Tax did not activate the CDK7 promoter in Jurkat cells (Figure 3B, right panel), suggesting that activation of the CDK7 promoter by Tax, in Kit 225 cells, is an indirect effect as a consequence of cell cycle progression. To explore the region of the *CDK7* gene, which directly responds to Tax, we cloned upstream (−2546 to −1732) and downstream (+51 to +815) regions of the *CDK7* gene upstream of an SV40 core promoter conjugated to a luciferase reporter gene. Neither the upstream nor the downstream region was activated by Tax in Jurkat cells (Figure 3B), and thus the direct Tax responsive element of the *CDK7* gene in Jurkat cells appears to reside in other regions of the promoter.

To gain further insight into the molecular mechanism of Tax-mediated activation of the CDK7 promoter in Kit 225 cells, we explored the signaling pathways mediating activation of the CDK7 promoter by Tax. Tax activates target genes through, at least in part, the NF-κB, CREB and SRF pathways. We thus examined the effect of Tax mutants, which lack the ability to activate each of those transcription factors, on activation of the CDK7 promoter. Tax mutant M22 lacks the ability to activate NF-κB (Figure 3C, right upper panel), d3 lacks the ability to activate CREB (Figure 3C, left lower panel) and 703 lacks the ability to activate SRF (Figure 3C, right lower panel). Note that each specific mutation not only lost the ability to activate its target transcription factor but also somewhat compromised activation of the other pathways. Tax mutant M22, which lacks the ability to activate the NF-κB pathway, could not activate the CDK7 promoter at all. In contrast, mutants lacking the ability to activate CREB (d3) or SRF (703) could activate the CDK7 promoter to some extent. These results suggest that the NF-κB pathway plays major roles in activation of the CDK7 promoter by Tax in Kit 225 cells.

### 3.4. The NF-κB Pathway Mediates Activation of the CDK7 Gene by Tax in Kit 225

**Figure 4:**
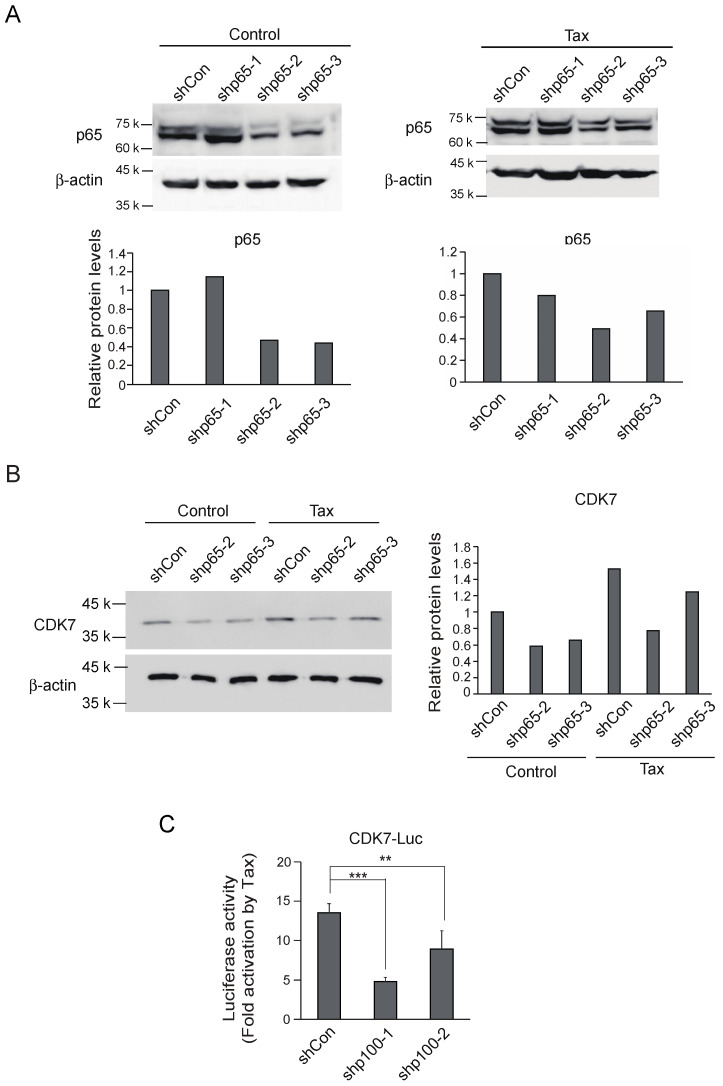
(**A**) Asynchronously growing Kit 225 cells were infected with recombinant adenovirus expressing shRNA against p65 or control virus along with Tax-expressing virus or control virus, further cultured for 3 days and harvested. Expression levels of p65 protein were examined by Western blot analysis (upper panels). The intensities of p65 bands were measured by ImageJ and adjusted by those of β-actin as an internal control (lower panels). (**B**) Under the same conditions, expression levels of CDK7 protein were examined by Western blot analysis except for shp65-1 (left panel). The intensities of CDK7 bands were measured by ImageJ and adjusted by those of β-actin as an internal control (right panel). (**C**) Asynchronously growing Kit 225 cells were transfected with CDK7 reporter along with expression vector for shRNA against p100 or control and Tax expression vector or control, further cultured for 3 days and harvested. Luciferase activities were measured and adjusted by those of β-galactosidase as an internal control. Fold activations by Tax are presented. **: 0.01 ≤ *p* < 0.05, ***: *p* < 0.01.

To confirm that activation of the *CDK7* gene in Kit 225 cells is mediated, at least in part, by the NF-κB pathway, we examined whether knockdown of NF-κB compromised activation of the *CDK7* gene by Tax. NF-κB is composed of three family members, RelA (p65 (RelA)/p50), RelB (RelB/p52) and c-Rel (c-Rel/p50). RelA and c-Rel are regulated by the classical pathway and RelB is regulated by the non-classical pathway. Among these, RelA (p65/p50) is the prototype of NF-κB. We generated expression vectors and recombinant adenovirus vectors for shRNA against p65 (shp65) at three different target sequences, and examined their effects in Kit 225 cells. Kit 225 cells were infected with the shp65-expressing adenovirus, with or without Tax-expressing adenovirus, cultured for 3 days in the absence of IL-2 and harvested. Expression levels of p65 were examined by Western blot analysis. Shp65-2 and shp65-3 successfully knocked down p65 expression, in the absence and presence of Tax (Figure 4A). We thus used both shp65-2 and shp65-3 constructs in further analyses, to exclude off-target effects. We next examined whether knockdown of p65 compromised induction of CDK7 expression by Tax by Western blot analysis in Kit 225 cells. Kit 225 cells were similarly infected with recombinant adenovirus expressing shp65-2, shp65-3 or control virus, with or without Tax-expressing virus, further cultured for 3 days in the absence of IL-2 and harvested. Knockdown of p65 reduced CDK7 protein expression both in the absence and presence of Tax (Figure 4B). To explore the potential involvement of the non-classical NF-κB pathway in Tax-mediated activation of CDK7 promoter, we examined whether knockdown of p100, the precursor of RelB partner p52, compromised activation of the CDK7 promoter in Kit 225 using the reporter assay. Effective knockdown of p100 by shRNA was demonstrated in our previous report [30]. Introduction of shRNAs against p100 compromised activation of the CDK7 promoter by Tax from about 13-fold to about 5- and 9-fold (Figure 4C). These results suggest that the non-classical NF-κB pathway mediates, at least in part, activation of the *CDK7* gene by Tax in Kit 225 cells. 

### 3.5. Induction of CDK7 Gene Expression Contributes to Tax-Mediated Gene Expression and Cell Cycle Progression

**Figure 5:**
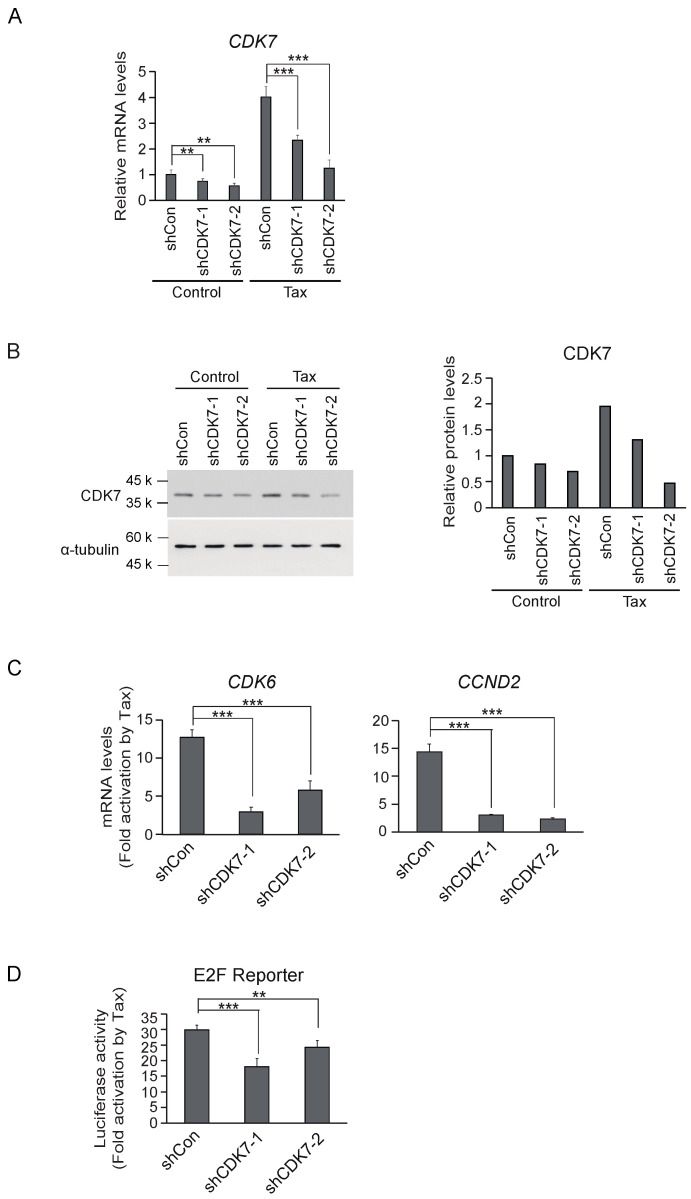
(**A**) Kit 225 cells were infected with recombinant adenovirus expressing shRNA against CDK7 along with Tax-expressing virus or control virus, cultured for 3 days and harvested. mRNA levels of *CDK7* were examined by qRT-PCR, adjusted by those of *GAPDH* as an internal control. Relative expression levels are presented. **: 0.01 ≤ *p* < 0.05, ***: *p* < 0.01. (**B**) Under the same conditions, the levels of CDK7 protein were examined by Western blot analysis (left panel). α-tubulin was used as an internal control. Intensities of CDK7 bands were measured by ImageJ and adjusted by those of α-tubulin (right panel). (**C**) Under the same conditions, the levels of *CCND2* and *CDK6* mRNAs were examined by qRT-PCR. Fold inductions by Tax are shown. ***: *p* < 0.01. (**D**) Asynchronously growing Kit 225 cells were transfected with E2F reporter plasmid along with expression vector for shRNA against CDK7 or control vector and expression vector for Tax or control vector, further cultured for 3 days and harvested. Luciferase activities were measured, adjusted by those of β-galactosidase as an internal control. Fold activations are shown. **: 0.01 ≤ *p* < 0.05, ***: *p* < 0.01.

Tax induces cell cycle progression, at least in part, by activating growth-related genes such as *CCND2* and *CDK6* in Kit 225 cells [30,31]. To explore the roles of CDK7 in Tax-mediated gene expression and cell cycle progression, we first examined whether induction of *CDK7* gene expression contributes to Tax-mediated gene expression. For this purpose, we examined whether shRNA-mediated knockdown of CDK7 expression reduced Tax-mediated activation of target genes. We generated expression vectors and recombinant adenoviruses for two shRNAs against CDK7 (shCDK7), to exclude off-target effects. We introduced shCDK7s into Kit 225 cells by recombinant adenovirus-mediated gene transfer and examined expression levels of *CDK7* mRNA by qRT-PCR. Both shCDK7s successfully downregulated *CDK7* mRNA expression in Kit 225 cells in the absence or presence of Tax (Figure 5A). We also examined expression levels of CDK7 protein by Western blot analysis. Both shCDK7s also downregulated expression of CDK7 protein in the absence or presence of Tax (Figure 5B). Using the same strategy, we also examined whether downregulation of Tax-induced CDK7 reduced Tax-mediated expression of *CCND2* and *CDK6* genes in Kit 225 cells. Both shCDK7s significantly reduced induction of *CCND2* and *CDK6* genes by Tax (Figure 5C), indicating that downregulation of CDK7 expression compromises Tax-mediated activation of cell cycle target genes.

Tax induces expression of growth-related genes and activates CDKs, leading to inactivation of RB and consequent activation E2F, which is essential for entry into the S phase and further cell cycle progression [30,31]. CDK7 contributes to both gene expression and activation of CDKs. Thus, it is predicted that Tax induction of CDK7 contributes to Tax-mediated activation of E2F and subsequent cell cycle progression. We thus explored whether downregulation of Tax-mediated expression of CDK7 suppressed Tax-mediated cell cycle progression. For this purpose, we first examined whether downregulation of CDK7 suppressed Tax-mediated activation of E2F, by a reporter assay. ShCDK7 expression vectors were co-transfected with Tax expression or control vectors, along with an E2F reporter plasmid, into Kit 225 cells, cultured in the absence of IL-2 for 3 days, and fold activation of the E2F reporter by Tax was examined. In the absence of shCDK7, Tax activated the E2F reporter by about 30-fold. In the presence of shCDK7-1 and shCDK7-2, Tax activation of the E2F reporter was significantly reduced to 18-fold and 24-fold, respectively (Figure 5D). These results suggest that downregulation of CDK7 expression suppressed Tax-mediated activation of E2F, and consequently inhibited Tax-mediated cell cycle progression. 

**Figure 6:**
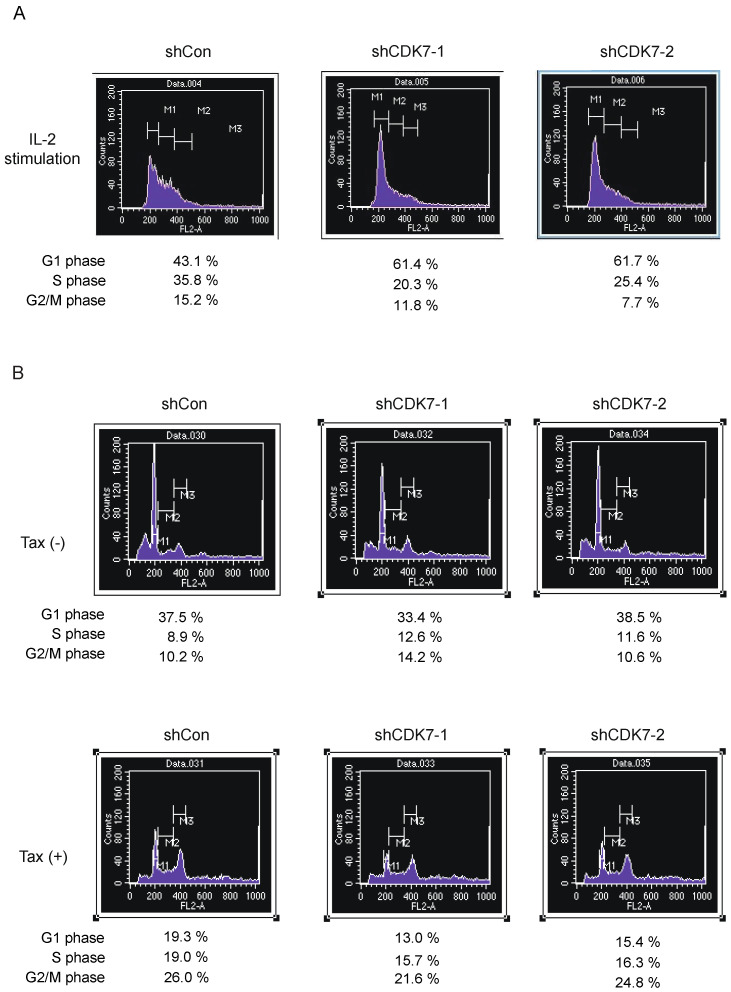
(**A**) Asynchronously growing Kit 225 cells were infected with recombinant adenovirus expressing shRNA against CDK7 or control virus, further cultured in the absence of IL-2 for 2 days, re-stimulated with IL-2 and harvested after 20 h. The cell cycle distribution of the cells was examined by FACS analysis of DNA content. (**B**) Asynchronously growing Kit 225 cells were infected with recombinant adenovirus expression shRNA against CDK7 or control virus along with Tax-expressing virus or control virus, further cultured in the absence of IL-2 for 3 days and harvested. The cell cycle distribution of the cells was determined by FACS analysis of the DNA content. The percentages of cells in G0/G1, S and G2/M are presented.

To confirm that downregulation of CDK7 expression suppressed Tax-mediated cell cycle progression, we examined cell cycle distribution by FACS analysis of the cellular DNA content. We first examined whether downregulation of CDK7 expression suppressed IL-2-mediated cell cycle progression as a positive control. Asynchronously growing Kit 225 cells were infected with shCDK7-expressing virus or control virus, starved of IL-2 for 2 days, re-stimulated with IL-2 and harvested 20 h after IL-2 stimulation. The DNA content of the cells was examined by FACS analysis after staining with propidium iodide. The percentage of cells in the S phase was reduced from 35.8% to 20.3% and 25.4%, respectively, suggesting that downregulation of CDK7 expression suppressed cell cycle progression induced by IL-2 (Figure 6A). We next examined whether downregulation of CDK7 expression similarly suppressed Tax-mediated cell cycle progression in Kit 225 cells, using shCDK7 constructs. Tax increased the percentage of cells in the S phase from 8.9% to 19.0% (Figure 6B). Co-expression of shCDK7-1 and shCDK7-2 with Tax decreased the fraction of cells in the S phase from 19.0% to 15.7% and 16.3%, respectively (Figure 6B), suggesting that downregulation of CDK7 expression suppresses Tax-mediated cell cycle progression.

## 4. Discussion

In this study, we identified the *CDK7* gene as a critical target of Tax, required for Tax-mediated activation of target genes and cell cycle progression. Tax induced CDK7 expression not only in human T-cell lines Kit 225 and Jurkat but also in normal PHA-PBLs. Tax activated the CDK7 promoter primarily via the NF-κB pathway, which mediates cell cycle progression by Tax in Kit 225. Moreover, downregulation of CDK7 expression suppressed Tax-induced target gene expression and cell cycle progression, indicating that Tax-mediated induction of CDK7 is an important component of Tax-dependent gene regulation and cell cycle progression. 

CDK7 is the catalytic component of CAK, which is composed of CDK7, CCNH and MAT1. CDK7 plays a crucial role in activation of CDKs by phosphorylating the T-loop of CDKs. For most CDKs, binding of cyclin alone is not sufficient for activation, and phosphorylation of the T-loop by CDK7 is also required [56,57]. Consistent with the role of CDK7 in cell cycle regulation, inhibition of CDK7 suppresses cell cycle progression [58,59]. We found that Tax induced *CDK7* gene expression and enhanced CDK7 protein expression along with phosphorylation of CDK2 at the site mediated by CDK7, suggesting the possibility that induction of CDK7 facilitates activation of cell-cycle-related CDKs by Tax. Our results showed that downregulation of CDK7 suppressed activation of E2F and cell cycle progression induced by Tax, suggesting that Tax-mediated induction of CDK7 is a critical element in Tax-dependent stimulation of cell cycle progression. 

CAK is also a catalytic component of TFIIH. CDK7 phosphorylates the C-terminal domain (CTD) of RNA polymerase II and facilitates the initiation of transcription. The C-terminal domain of RNA polymerase II is comprised of 52 repeats of 7 amino acids (Tyr-Ser-Pro-Thr-Ser-Pro-Ser) and is thought to function as an anchor at transcription initiation sites. CDK7 phosphorylates the fifth and likely the seventh Ser residues in these heptad repeats to release the CTD from transcription initiation sites, thereby facilitating transcription initiation [57]. Indeed, downregulation of CDK7 by shRNA reduced Tax-mediated induction of *CCND2* and *CDK6* genes, supporting the notion that induction of CDK7 expression by Tax contributes to Tax-mediated cell cycle target gene expression. RNA polymerase II released from anchoring at the transcriptional start sites still pauses at promoter proximal sites [60,61]. This pause is released, at least in part, by phosphorylation of the CTD at Ser2 by CDK9/CCNT, which together form the Positive Transcription Elongation Factor b (P-TEFb), resulting in productive elongation. We also found that Tax increased expression of the *CDK9* and *CCNT* genes, suggesting that Tax also facilitates transcription by reducing the RNA polymerase II pause at promoter proximal sites. Phosphorylation of the T-loop of CDK9 by CDK7 is also required for activation of CDK9/CCNT [62]. This suggests that induction of CDK7 by Tax may also contribute to Tax-mediated transcription by enhancing CDK9/CCNT activity.

Although Tax induced *CDK7* gene expression both in Kit 225 and Jurkat cells, Tax activated the CDK7 promoter in Kit 225, but not in Jurkat cells. This suggests that the direct Tax response element resides in another region than the CDK7 promoter region we analyzed and that activation of the CDK7 promoter is an indirect effect as a consequence of cell cycle progression in Kit 225. Indeed, activation of the CDK7 promoter by Tax in Kit 225 was dependent on the NF-κB pathway, which is required for Tax-mediated cell cycle progression in Kit 225 [30,31]. These observations suggest the presence of a transcription factor, which is activated concomitant with cell cycle progression and contributes to activation of the *CDK7* gene. A direct Tax response element in the *CDK7* gene and a transcription factor, which is activated by cell cycle progression and activates the *CDK7* gene, remain to be identified.

Knockdown of p65 reduced CDK7 expression, not only in the presence of Tax but also in the absence of Tax, in Kit 225 cells (Figure 4B). We speculate that this could be due to constitutive activation of NF-κB in Kit 225 cells. Our results suggest that, although Tax can activate an NF-κB reporter in Kit 225 cells, the degree of activation is smaller than that in Jurkat cells. Moreover, Kit 225 cells can be arrested in G0 by deprivation of IL-2, without apparent apoptosis, in contrast to most immune cells, which are susceptible to apoptosis upon withdrawal of growth factors. This may be due to constitutive activation of NF-κB in Kit 225 cells, to maintain viability, which may also contribute to expression of CDK7 in the absence of IL-2, and could underly the downregulation of CDK7, by knockdown of p65, in the absence of Tax.

In contrast to the significant induction of CDK7, Tax minimally increased expression of CCNH, the cyclin partner of CDK7. It is generally accepted that expression of CDKs is relatively constitutive and that their activity is regulated by cyclic induction and destruction of their obligate cyclin partners. Although expression of CCNH was scarcely induced by Tax, Tax enhanced phosphorylation of CDK2 and the CTD of RNA polymerase II, which are known to be mediated by CDK7, concomitant with evident induction of CDK7 expression. This suggests the possibility that, in contrast to the functional relationship of other CDKs and cyclins, the activity of CDK7/CCNH may be regulated primarily by expression of CDK7 alone and not by CCNH. To approach this possibility, we attempted to overexpress CDK7, using recombinant adenovirus expressing CDK7 under the control of the CMV promoter, to determine if phosphorylation of CDK2 and CTD was enhanced. However, we were unable to obtain a significant level of CDK7 expression, possibly due to the extremely low activity of the CMV promoter in Kit 225 cells, as indicated by the minimal activity of the CMV-β-galactosidase construct, used as an internal control in reporter assays. Thus, the impact of isolated overexpression of CDK7 on CDK7/CCNH kinase activity remains to be determined.

Accumulating evidence indicates that CDK7 is often overexpressed, and its activity is enhanced in a variety of cancers, thereby contributing to aberrant proliferation of cancer cells [63,64]. These observations identified CDK7 as a potential target of interest for cancer therapy, and small molecule inhibitors of CDK7 are currently under development [57,64,65,66,67,68,69]. Herein, we showed that Tax induced CDK7 expression, and demonstrated that knockdown of CDK7 attenuated Tax-dependent activation of target gene transcription and cell cycle progression, suggesting that CDK7 may also serve as an important therapeutic target molecule in the treatment of ATL.

## Data Availability

The raw data supporting the conclusions of this article will be made available by the authors on request.
